# Novel Respiratory Syncytial Virus Subtype ON1 among Children, Cape Town, South Africa, 2012

**DOI:** 10.3201/eid1904.121465

**Published:** 2013-04

**Authors:** Ziyaad Valley-Omar, Rudzani Muloiwa, Nai-Chung Hu, Brian Eley, Nei-Yuan Hsiao

**Affiliations:** Author affiliations: University of Cape Town, Cape Town, South Africa (Z. Valley-Omar, R. Muloiwa, B. Eley, N.-Y. Hsiao);; National Institute for Communicable Diseases, Cape Town (Z. Valley-Omar, N.-C. Hu, N.-Y. Hsiao);; Red Cross War Memorial Children’s Hospital, Cape Town (R. Muloiwa, B. Eley);; National Health Laboratory, Cape Town (N.-Y. Hsiao)

**Keywords:** Respiratory syncytial virus, nosocomial, subtype, genotype, ON1, NA1, pediatric, RSV, viruses, Cape Town, South Africa

**To the Editor**: Human respiratory syncytial virus (RSV) is a common cause of severe acute lower respiratory tract infection in young children, accounting for ≈160,000 deaths/year worldwide (*1**,**2*). As part of an RSV nosocomial transmission study, we detected RSV genotype ON1, which was identified during November 2010­–February 2011 as a novel genotype in Ontario, Canada, in samples from children in a tertiary pediatric hospital in Cape Town, South Africa during 2012. The genotype described in Canada was characterized by a 72-nt sequence duplication within the second variable domain of the envelope glycoprotein. The 72-nt duplication within the second variable domain in ON1 was the largest sequence duplication described in this virus (*3*).

RSV is divided into 2 genetically distinct groups, RSV A and B, based on the viral envelope glycoprotein nucleotide sequences (*4*). Sequence variability in the C-terminal variable domain of the glycoprotein gene is commonly used to determine RSV phylogeny (*3**,**5*). To date, 11 RSV A (ON1, GA1–GA7, SAA1, NA1, and NA2) and 17 RSV B (GB1–GB4, SAB1–SAB3, and BA1–BA10) genotypes have been identified (*3**,**6*).

As part of the aforementioned molecular epidemiology study surveying RSV infection in a pediatric hospital, (University of Cape Town research ethics study no. 305/2012), we sequenced the RSV glycoprotein second variable domain of nucleic acid extracts derived from RSV-positive respiratory secretion samples from 160 young children hospitalized for treatment of respiratory tract infections. The techniques used have been described (*7*). During January–April, in an area where NA1 was the dominant circulating RSV genotype, 119 (74%) of 160 RSV isolates were RSV A. We noted the presence, albeit at a low incidence, of the novel ON1 genotype cluster (8 viral isolates) (Technical Appendix) in specimens collected during February–April.

Children in the RSV ON1-infected cohort were brought to health care facilities during February 24–April 25, 2012 (Figure and Technical Appendix), where they received a diagnosis of brochiolitis or bronchiopneumonia (Technical Appendix). With the exception of 1 patient, child 8, who had been hospitalized before onset of this illness, all ON1 isolates were community acquired. Seven of the 8 ON1 isolates were obtained from infants <4 months of age (median 7 weeks), who were younger than the 152 children who were not infected with the ONI genotype (median age 3.5 months). The RSV ON1–infected children lived within a 2.5-km radius of one another (Technical Appendix). The children who were not infected with RSV ON1 lived in a much wider geographic area; >90% lived within an 18-km radius of one another. These spatial associations with disease prevalence suggested that the ON1-infected children represented a localized cluster of transmission.

**Figure:**
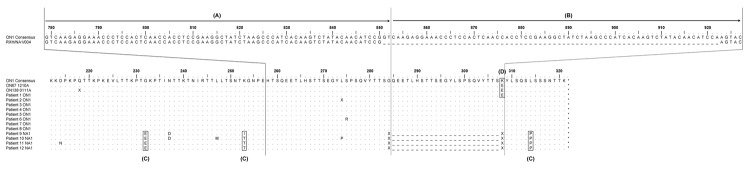
Alignment of deduced amino acid sequences for ON1 isolates from South Africa (Patient 1–8, accession nos. JX885730–JX885737) and Canada (ON67 and ON138) with NA1 isolates (Patient 9–12) from South Africa. A) Variable domain sequence copied, B) Duplicated sequence inserted into variable domain, C) Characteristic amino acid substitutions that distinguish ON1 from NA1. D) Amino acid substitution (E308K) (position 284 before insertion) that distinguishes between most ON1 isolates from South Africa (Patient 2–8) from those from Canada (ON67 and ON138) ON1.

None of the children were infected with HIV, although 3 had antenatal exposure to HIV. Co-infection with adenovirus and rhinovirus was noted in 3 of the patients. Although 3 of the patients were hospitalized for a prolonged period and required ventilatory support, the severity and outcome of the RSV ON1 infections were similar to RSV infections caused by other genotypes among children of the same age.

Sequence analyses revealed that ON1 isolates identified in South Africa are essentially identical to those isolated in Canada, possessing characteristic amino acid substitutions at positions E232G, T253K, and P314L that distinguish the genotype from circulating NA1 genotypes (*3*). However, 7 of 8 ON1 isolates from South Africa possess a unique E308K (position 284 before insertion) amino acid change at the 3′ border of the duplicated gene segment not present in the ON1 isolates identified in Canada (Figure). The conservation of the E308K mutation within ≈90% of the isolates from South Africa that we studied suggests a possible functional role for the positively charged lysine residue.

The capacity of the RSV glycoprotein to accommodate large insertions and remain functional was first demonstrated with the RSV B, BA genotype (Buenos Aires, Argentina 1999). This genotype contains a 60-nt duplication in the second variable domain, which, similar to ON1, did not cause serious clinical outcomes (*6**,**8**–**10*). Longitudinal analyses during 12 epidemic seasons (1996–97 through 2007–08) of international RSV subtype distribution revealed that since its initial detection in 1999, BA prevalence has gradually increased to become the dominant RSV group B virus genotype in circulation (*10*). Because RSV A has traditionally been the dominant RSV type in circulation, if the large insertion in ON1 confers similar selection advantage as seen in BA, the potential dominance of a single ON1 genotype within this group might promote bias on RSV type distribution toward RSV A.

The novel ON1 genotype was first described in Ontario, Canada (*3*). Our subsequent findings in South Africa suggest extensive distribution of this genotype, which was assumed to have arisen before winter 2010–11 (*3*). To understand whether ON1 in South Africa occurred as a result of importation or natural evolution within locally circulating NA1 genotypes, further research is required.

Technical Appendix Respiratory syncytial virus cases among hospitalized children and epidemiologic observations for patients infected with respiratory syncytial virus ON1 in a hospital pediatric unit, Cape Town, South Africa.
