# Supplementary material for: Novel Respiratory Syncytial Virus Subtype ON1 among Children, Cape Town, South Africa, 2012
Source: Emerg Infect Dis. 2013 Apr;19(4):668–70. doi: 10.3201/eid1904.121465 (PMC3647422; doi:10.3201/eid1904.121465)
Supplement: Technical Appendix — Respiratory syncytial virus cases among hospitalized children and epidemiologic observations for patients infected with respiratory syncytial virus ON1 in a hospital pediatric unit, Cape Town, South Africa. [file 12-1465-Techapp-s1.pdf]

Article DOI: http://dx.doi.org/10.3201/eid1904.121465

# Novel Respiratory Syncytial Virus Subtype ON1 among Children, Cape Town, South Africa, 2012

## Technical Appendix

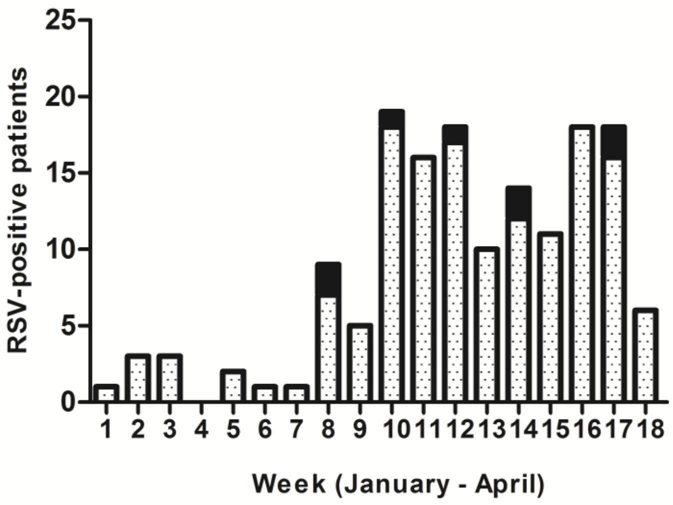


Technical Appendix Figure. Respiratory syncytial virus (RSV) cases among hospitalized children, Cape Town, South Africa. Stippled bar sections indicate non-ON1 genotype infections. Black bar sections indicate ON1 genotype infections.

Technical Appendix Table. Epidemiologic observations for patients infected with respiratory syncytial virus ON1 in a hospital pediatric unit, Cape Town, South Africa*

| Pt no. | Sex/age, wk | LOS, d | HIV exposure/status | ICU/HDU care | Respiratory support | Discharge diagnosis | Concurrent conditions | Co-infection |
|---|---|---|---|---|---|---|---|---|
| 1 | F/15 | 2 | U/– | No | No | Bronchial pneumonia | Preterm birth | None |
| 2 | M/1.5 | 1 | U/– | No | No | Bronchiolitis | Neonatal jaundice | None |
| 3 | F/4 | 7 | U/– | HDU | CPAP | Bronchiolitis | None | None |
| 4 | F/3 | 1 | E/– | No | No | Bronchiolitis | None | Adenovirus |
| 5 | F/10 | 2 | E/– | No | No | Lobar pneumonia | Neonatal sepsis | Rhinovirus |
| 6 | F/8 | 38 | U/– | ICU | IPPV | Bronchiopneumonia | Preterm birth | None |
| 7 | F/6.5 | 12 | E/– | HDU | CPAP | Bronchiopneumonia | Preterm birth | None |
| 8 | M/60 | 1 | U/– | No | No | Bronchiolitis obliterans | Recurrent LRTI after adenovirus infection | Rhinovirus |

*Pt, patient; LOS, length of stay in hospital; ICU, intensive care unit; HDU, high dependency unit; U, unexposed; –, negative; E, exposed; LRTI, lower respiratory tract infection; CPAP, continuous positive airway pressure; IPPV, intermittent positive pressure ventilation.
